# Phosphorylation of Serine 248 of C/EBPα Is Dispensable for Myelopoiesis but Its Disruption Leads to a Low Penetrant Myeloid Disorder with Long Latency

**DOI:** 10.1371/journal.pone.0038841

**Published:** 2012-06-08

**Authors:** Marie S. Hasemann, Mikkel B. Schuster, Anne-Katrine Frank, Kim Theilgaard-Mönch, Thomas Å. Pedersen, Claus Nerlov, Bo T. Porse

**Affiliations:** 1 The Finsen Laboratory, Rigshospitalet, Faculty of Health Sciences, University of Copenhagen, Copenhagen, Denmark; 2 Biotech Research and Innovation Center (BRIC), University of Copenhagen, Copenhagen, Denmark; 3 Danish Stem Cell Centre (DanStem) Faculty of Health Sciences, University of Copenhagen, Copenhagen, Denmark; 4 Deptartment of Hematology, Skanes University Hospital, University of Lund, Lund, Sweden; 5 European Molecular Biology Laboratory (EMBL) Mouse Biology Unit, Monterotondo, Italy; 6 Medical Research Council (MRC) Center for Regenerative Medicine, Institute for Stem Cell Research, University of Edinburg, Edinburg, United Kingdom; Georg Speyer Haus, Germany

## Abstract

**Background:**

Transcription factors play a key role in lineage commitment and differentiation of stem cells into distinct mature cells. In hematopoiesis, they regulate lineage-specific gene expression in a stage-specific manner through various physical and functional interactions with regulatory proteins that are simultanously recruited and activated to ensure timely gene expression. The transcription factor CCAAT/enhancer binding protein α (C/EBPα) is such a factor and is essential for the development of granulocytic/monocytic cells. The activity of C/EBPα is regulated on several levels including gene expression, alternative translation, protein interactions and posttranslational modifications, such as phosphorylation. In particular, the phosphorylation of serine 248 of the transactivation domain has been shown to be of crucial importance for granulocytic differentiation of 32Dcl3 cells *in vitro*.

**Methodology/Principal Findings:**

Here, we use mouse genetics to investigate the significance of C/EBPα serine 248 *in vivo* through the construction and analysis of *Cebpa* ^S248A/S248A^ knock-in mice. Surprisingly, 8-week old *Cebpa* ^S248A/S248A^ mice display normal steady-state hematopoiesis including unaltered development of mature myeloid cells. However, over time some of the animals develop a hematopoietic disorder with accumulation of multipotent, megakaryocytic and erythroid progenitor cells and a mild impairment of differentiation along the granulocytic-monocytic lineage. Furthermore, BM cells from *Cebpa* ^S248A/S248A^ animals display a competitive advantage compared to wild type cells in a transplantation assay.

**Conclusions/Significance:**

Taken together, our data shows that the substitution of C/EBPα serine 248 to alanine favors the selection of the megakaryocytic/erythroid lineage over the monocytic/granulocytic compartment in old mice and suggests that S248 phosphorylation may be required to maintain proper hematopoietic homeostasis in response to changes in the wiring of cellular signalling networks. More broadly, the marked differences between the phenotype of the S248A variant *in vivo* and *in vitro* highlight the need to exert caution when extending *in vitro* phenotypes to the more appropriate *in vivo* context.

## Introduction

The human body contains trillions of blood cells that are continuously replaced through normal cell turnover. Hematopoiesis is the highly orchestrated process responsible for regulating the generation of mature blood cells from a rare population of hematopoietic stem cells (HSC). The HSCs possess the ability to self-renew and differentiate into all blood lineages and are the ultimate reservoir for maintaining the supply of blood cells throughout life. Multiple mechanisms are required in order to meet both the changing demands from the body and to maintain steady-state hematopoiesis [1]. In particular, many transcription factors have been shown to modulate key events in differentiation and proliferation and their function in hematopoiesis has been investigated thoroughly through the examination of knockout mice [2]. One of these transcription factors is CCAAT/enhancer binding protein alpha (C/EBPα), which is not only involved in regulation of hematopoiesis, but also exerts its function in other tissues such as lung, liver and adipose tissue through the induction of lineage-specific gene programs in combination with an ability to promote cell cycle exit [3], [4], [5]. Within the hematopoietic system, C/EBPα has been shown to be important for the myeloid lineage, since conditional deletion of the *Cebpa* allele in the hematopoietic compartment of adult mice blocks the transition from common myeloid progenitors (CMPs) to granulocyte-monocyte progenitors (GMPs), thus resulting in complete loss of granulocytes, monocytes and eosinophils [6], [7]. Besides this late granulocytic-monocytic differentiation block, fetal livers of newborn *Cebpa null* mice display increased numbers of progenitors and mature cells of the erythroid lineage, suggesting that C/EBPα might play a role in repressing erythroid differentiation [7]. In line with this, overexpression of C/EBPα in erythroid progenitor cells, redirects the differentiation potential in a granulocytic direction resulting in an increased level of mature granulocytes and granulocyte-monocyte progenitors with a concomitant decrease of erythroid progenitors [8].

The activity of C/EBPα is tightly controlled through multiple layers of regulation. First of all, timely expression is required and involves regulation of gene transcription, mRNA translation and protein degradation [9], [10]. Secondly, protein interactions have a major impact on the ability of C/EBPα to induce or repress gene transcription [11], [12], [13]. Thirdly, C/EBPα activity can be altered by posttranslational modifications such as sumyolation and phosphorylation [14], [15]. The phosphorylation status of serine 21 (S21) has been shown to have a major impact on the decision to differentiate towards the monocytic or granulocytic lineage *in vitro*. Upon phosphorylation of S21 or expression of a phospho-mimicking mutant in K-562 cells, granulopoiesis is inhibited, thereby favoring monocytic differentiation at the cost of granulocytic differentiation [15], [16]. Furthermore, the phosphorylation of serine 248 in the transactivation domain has been suggested to be required for myeloid differentiation since mutating it to an alanine residue abrogates the capacity of C/EBPα to induce granulocytic differentiation of 32Dcl3 cells *in vitro* [17]. Serine 248 (S248) is phosphorylated by activated Ras signaling and this phosphorylation increases the ability of C/EBPα to promote expression from the G-CSF receptor promoter. Therefore, it appears that phosphorylation and dephosphorylation of C/EBPα at distinct serine residues can directly push the cells towards a specific myeloid branch. However, these phosphorylation sites have only been investigated in an *in vitro* context and what the functions are *in vivo* is therefore unknown.

We and others have previously reported on several *Cebpa* knock-in mouse models [18], [19], [20], [21], [22], [23], which have provided valuable information pertaining the role of C/EBPα in myeloid differentiation and in the development of leukemia. In this study, we use knock-in mutagenesis to elucidate the importance of S248 phosphorylation for myeloid differentiation by introducing an allele of *Cebpa* with an alanine substituted for serine 248, thereby abrogating phosphorylation of this residue. Surprisingly, we could show that whereas myeloid differentiation of cells expressing C/EBPα-S248A is blocked *in vitro*, this is not the case *in vivo*. Thus, young *Cebpa* ^S248A/S248A^ mice display no phenotypic alterations in the hematopoietic compartment or other tissues. In contrast, aged *Cebpa* ^S248A/S248A^ animals develop a low-penetrant myeloid disorder characterized by a mild impairment of differentiation along the granulocytic-monocytic lineage and by the accumulation of HSCs, multipotent progenitor cells (MPPs), as well as megakaryocytic and early erythroid progenitors.

## Results

### S248 is required for C/EBPα to promote granulocytic differentiation in vitro

**Figure 1:**
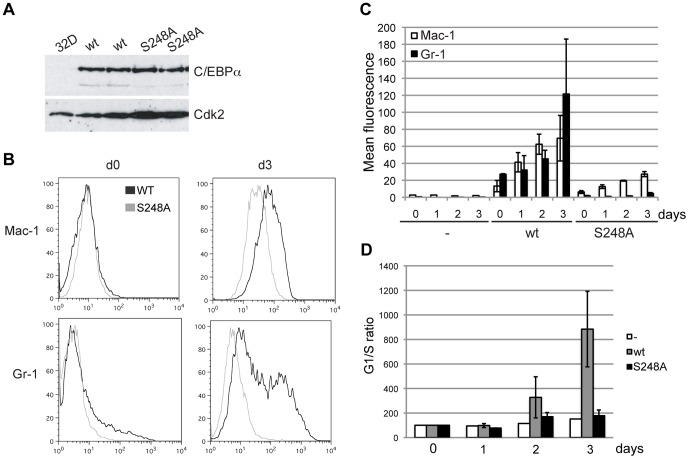
C/EBPα-S248A cannot induce differentiation of 32Dcl3 cells. (A) Western blot analysis of 32Dcl3 clones expressing C/EBPα-ER (wt) or C/EBPα-S248A-ER (S248A). (B) Flow cytometry analysis of expression of the granulocytic-monocytic markers Mac-1 and Gr-1 following 4-OHT addition to the C/EBPα-ER or C/EBPα-S248A-ER expressing clones. (C) Quantification of the flow cytometry data in (B) from two experiments using two independent wt-ER and S248A-ER clones (mean +/− standard deviation). (D) Quantification of the G1/S ratios determined by flow cytometry of BrdU and Propidium Iodide stained cell cultures. The data is from two experiments (mean +/− standard deviation).

The murine myeloid 32Dcl3 cell line has long been considered a suitable *in vitro* model system for analyzing myelopoiesis, since it is one of the few cell lines that can terminally differentiate into mature neutrophils. The cell line is diploid and non-leukemic in syngenic murine recipients [24], [25]. It proliferates in media containing IL-3 however, upon removal of this cytokine and addition of G-CSF, proliferation ceases and differentiation into neutrophil granulocytes proceeds. It is well documented that ectopic expression of C/EBPα in 32Dcl3 cells is sufficient to induce terminal granulocytic differentiation even in the presence of IL-3, making this a suitable differentiation assay to analyze the effect of C/EBPα mutations on this process [24], [25]. In order to investigate if C/EBPα-S248A is defective in granulocytic-monocytic *in vitro* differentiation as previously reported [17], 32Dcl3 clones expressing either a wild type C/EBPα-estrogen receptor ligand-binding domain fusion protein (C/EBPα-ER) or the C/EBPα-S248A-ER variant were constructed and clones expressing an equal amount of protein were selected for further analysis (Figure 1A).

To test whether S248 is required for the ability of C/EBPα to promote granulocytic differentiation C/EBPα-ER was translocated to the nucleus by addition of 4-hydroxytamoxifen (4-OHT). Cells were monitored for three days, and samples were collected each day and analyzed by flow cytometry to assess proliferation (Figure 1D) and granulocytic differentiation (Figure 1B, C). As expected 32Dcl3-C/EBPα-ER cells exited cell cycle within a few days, the G1/S ratio increased 15 fold (Figure 1D) and cells became positive for the granulocytic-monocytic marker Mac-1 and later for Gr-1 (Figure 1B, C). On the other hand, nuclear translocation of C/EBPα-S248A-ER did not lead to growth arrest and cells failed to express the two differentiation markers.

Thus in agreement with previous findings [17], these data show that S248 is necessary for C/EBPα to induce growth arrest and differentiation of neutrophil granulocytes *in vitro*.

### Initial analysis of *Cebpa* ^S248A/S248A^ knock-in mice

In order to investigate the importance of C/EBPα-S248 *in vivo*, we generated a knock-in mouse line, in which the wild type *Cebpa* gene was replaced with an allele expressing C/EBPα-S248A. *Cebpa* ^S248A/S248A^ knock-in mice were born and weaned in Mendelian ratios, were physically indistinguishable from both wild type littermates and the more appropriate *Cebpa* ^KI/KI^ controls [20], [26], showed no visible signs of illness and were fully fertile (data not shown), thus demonstrating that S248 of C/EBPα is fully dispensable for embryonic survival. Furthermore, inspection of tissues in which C/EBPα function has previously been demonstrated to be important, such as liver, lung, spleen and white adipose tissue revealed no obvious abnormalities neither in terms of morphology nor size in the *Cebpa* ^S248A/S248A^ animals (Figure S1 and data not shown). These findings suggest that S248 is dispensable for the development and maintenance of these tissues.

### Young *Cebpa* ^S248A/S248A^ mice are phenotypically normal in the hematopoietic system

**Figure 2:**
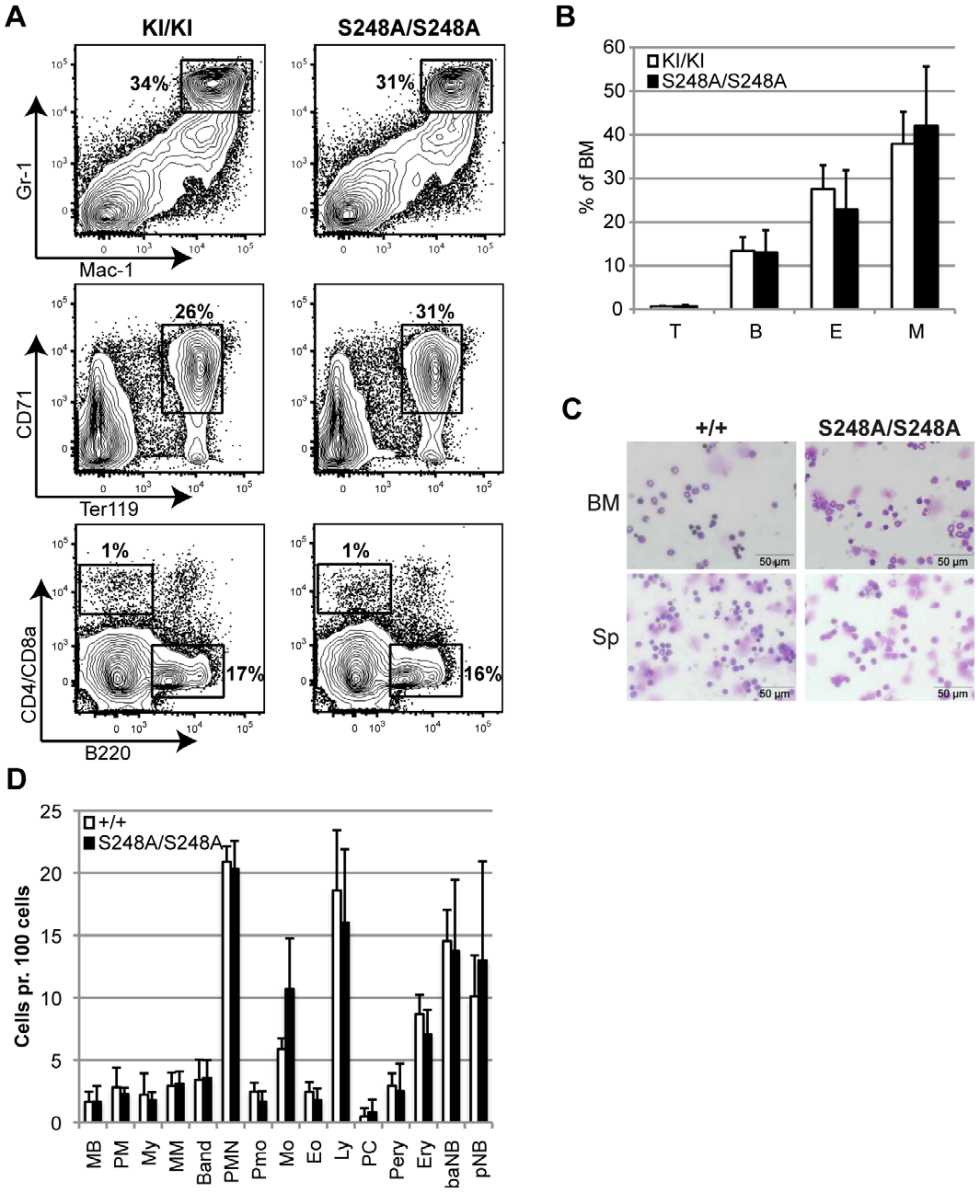
Young *Cebpa* ^S248A/S248A^ mice display no overt hematopoietic phenotype. (A) The mature hematopoietic lineages in the BM from 8 week-old *Cebpa* ^KI/KI^ and *Cebpa* ^S248A/S248A^ mice were analyzed by flow cytometry using cell surface-specific markers for neutrophil granulocytes (M = Mac-1+, Gr-1+), erythroid cells (E = CD71+, Ter119+), B cells (B = B220+) and T cells (T = CD4+ or CD8a+). (B) Quantification of the data from (A). *Cebpa* ^KI/KI^ (n = 6) and *Cebpa* ^S248A/S248A^ (n = 6) (mean +/− standard deviation). (C) Cytospin analysis of BM and spleen cells from *Cebpa* ^+/+^ and *Cebpa* ^S248A/S248A^ mice. (D) Differential counts of the cytospins in (C) from *Cebpa* ^+/+^ (n = 3) and *Cebpa* ^S248A/S248A^ (n = 4) (mean +/− standard deviation). Abbreviations indicate the granulocytic (Myeloblasts (MB); Promyelocytes (PM); Myelocytes (My); Metamyelocytes (MM); Band cells (Band) and Polymorphonuclear Granulocytes (PMN)) monocytic (Promonocytes (Pmo); Monocytes (Mo)), erythroid (Proerythrocytes (Pery); Erythrocytes (ery); basophilic normoblast (bNB); polychromatic normoblast (pNB)); lymphocytes (Ly); eosinophils (Eo) and plasma cell (PC) types.

C/EBPα is a key regulator of myeloid differentiation and altered C/EBPα activity has been shown to affect hematopoietic development and predispose to myeloid malignancies such as AML [3], [21], [22]. Moreover, the finding that C/EBPα-S248A was unable to direct granulocytic-monocytic differentiation *in vitro* prompted us to investigate the hematopoietic system of *Cebpa* ^S248A/S248A^ mice. Bone marrows (BM) from 8 week-old *Cebpa* ^S248A/S248A^ and *Cebpa* ^KI/KI^ mice were collected and stained with antibodies for cell-surface markers specific for the different mature lineages and analyzed by flow cytometry. Surprisingly, in contrast to the *in vitro* data reported above, mutation of S248 did not alter the frequency of mature granulocytes (Mac-1+, Gr-1+) and the prevalence of other BM populations such as B cells (B220+), T cells (CD4+ and/or CD8+), erythroid cells (Ter119+, CD71+/int) was also unaffected (Figure 2A, B). Furthermore, examination of cytospins prepared from BM or spleen from 8 week-old mice showed no aberrations in cellular morphologies or distributions (Figure 2C, D). These data suggest, that S248 of C/EBPα is dispensable for the *in vivo* development of mature hematopoietic lineages.

**Figure 3:**
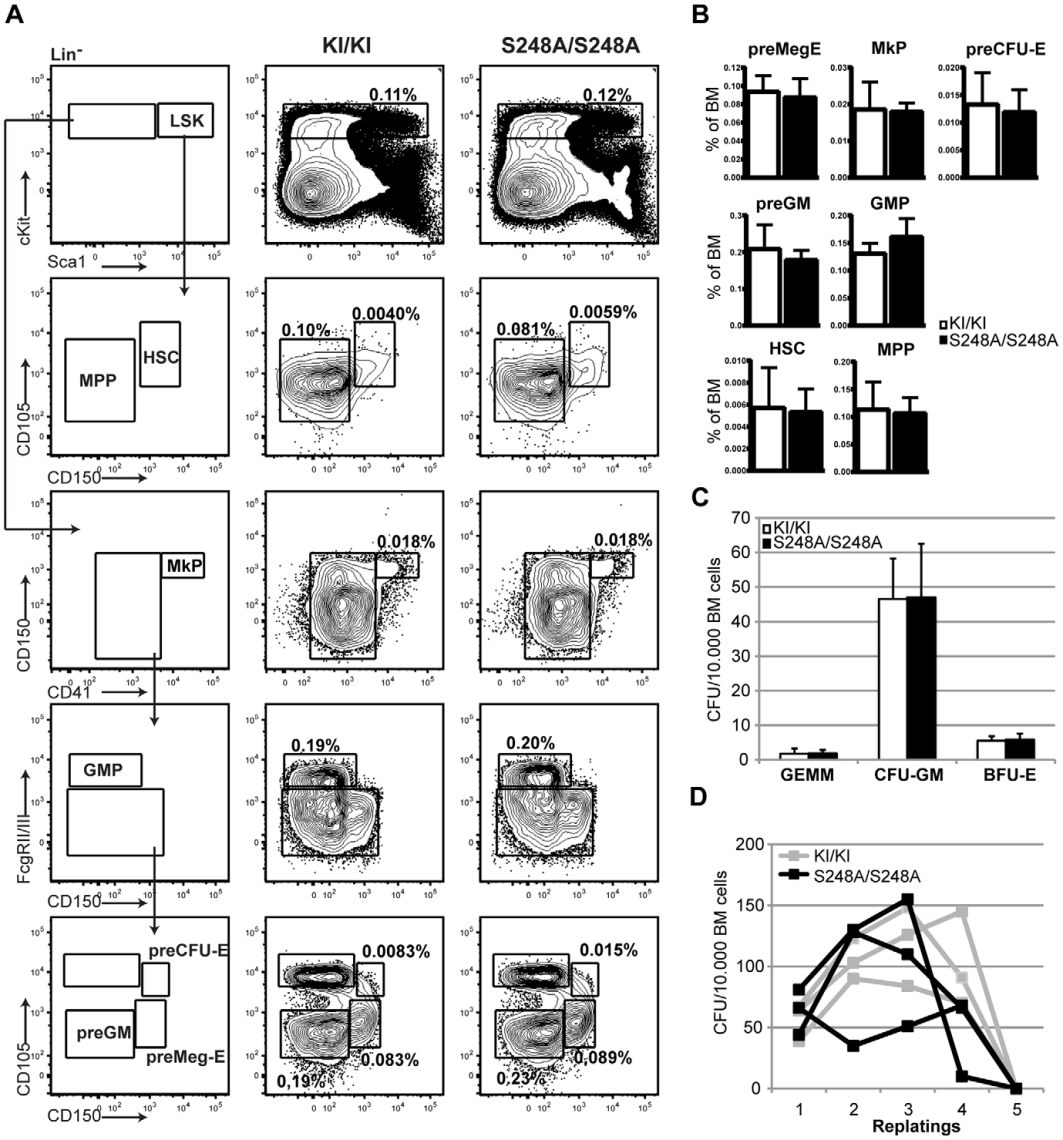
Young *Cebpa* ^S248A/S248A^ mice show no abnormalities in the hematopoietic stem and progenitor compartment. (A) Flow cytometry analysis of the stem and progenitor compartment in the BM from *Cebpa* ^KI/KI^ and *Cebpa* ^S248A/S248A^. (B) Quantification of the data in (A). There were no significant changes in *Cebpa* ^KI/KI^ (n = 6) and *Cebpa* ^S248A/S248A^ (n = 6) mice (mean +/− standard deviation). (C) Colony forming unit assays of BM cells from *Cebpa* ^KI/KI^ (n = 4) and *Cebpa* ^S248A/S248A^ (n = 4) mice (mean +/− standard deviation). (D) Serial replating of the primary colonies from (C) showing the behavior of 3 individual mice of each genotype.

Since *Cebpa null* mice have previously been shown to accumulate myeloid progenitors and to harbor a differentiation block upstream of the GMP [6], we analyzed the myeloid progenitor compartment using the antibody panel reported by Pronk et al. [27]. However, we were unable to detect any major alterations in the cellular distributions of myeloid progenitors or HSCs/MPPs (Figure 3A, B), suggesting that S248 of C/EBPα is not required for steady state lineage commitment or differentiation of myeloid progenitors in young mice.

To evaluate the myeloid progenitors functionally, we next plated BM cells from 8 week-old *Cebpa* ^S248A/S248A^ and *Cebpa* ^KI/KI^ animals in semisolid media to allow for the outgrowth of all myeloid colonies. In contrast to previous studies involving BM progenitors derived from other *Cebpa* mutant mouse lines [19], [21], *Cebpa* ^S248A/S248A^ BMs yielded similar distributions of BFU-E, CFU-GM and CFU-GEMM colonies as the *Cebpa* ^KI/KI^ controls (Figure 3C). Furthermore, when we analyzed the proliferative consequences of mutating S248 by performing serial replating of colonies derived from the first round of plating, *Cebpa* ^S248A/S248A^ progenitors displayed the same limited proliferative potential as progenitors from the *Cebpa* ^KI/KI^ controls when counted either as colonies (Figure 3D) or as total cell number (data not shown).

In conclusion, at eight weeks of age *Cebpa* ^S248A/S248A^ mice display no overt phenotype in the hematopoietic compartment.

### A fraction of *Cebpa* ^S248A/S248A^ mice develop a low-penetrant hematopoietic disorder with long latency

Many hematopoietic diseases are particularly prevalent in the elderly population, but the mechanisms involved are not resolved but may be related to an increase in myeloid-based HSCs upon ageing or accumulation of acquired genetic mutations [28]. We therefore analyzed a cohort of *Cebpa* ^S248A/S248A^ animals at one year of age (12–14 months) to detect whether mutation of S248 led to any phenotypic changes in the hematopoietic compartment in older animals. At this age, several of the *Cebpa* ^S248A/S248A^ mice (4/23) had enlarged spleen (Figure S2D), whereas all of the knock-in wild type controls were phenotypically indistinguishable from the young wild type controls.

**Figure 4:**
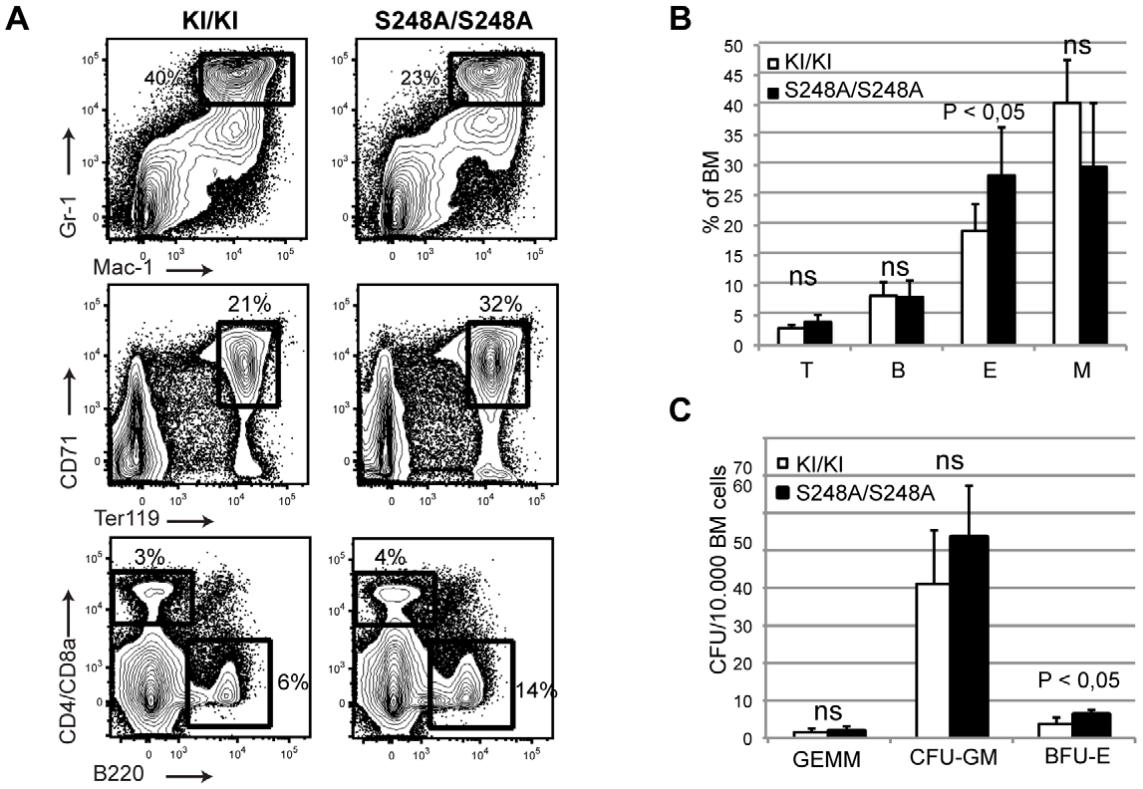
One-year old *Cebpa* ^S248A/S248A^ mice display an erythroid lineage bias. (A) Flow cytometry analysis of mature populations in BMs from *Cebpa* ^KI/KI^ (n = 6) and *Cebpa* ^S248A/S248A^ (n = 6) mice. Analyzed populations: Erythroid cells (E = CD71+, Ter119+), granulocytes (M = Mac-1+, Gr-1+); B-cell (B = B220+) and T-cells (T = CD4+ or CD8a+). (B) Quantification of the data from (A). (C) Colony forming unit assays of BM cells from *Cebpa* ^KI/KI^ (n = 4) and *Cebpa* ^S248A/S248A^ (n = 5) mice (mean +/− standard deviation).

Next, we analyzed the distribution of the mature hematopoietic populations within the BM of cohorts of *Cebpa* ^KI/KI^ control and *Cebpa* ^S248A/S248A^ mice. In the BM from *Cebpa* ^S248A/S248A^ mice there was a slight reduction in mature neutrophil granulocytes (Mac-1+, Gr-1+) and a concomitant increase in erythroid progenitors (Ter119+, CD71+), whereas the lymphoid compartment was unaffected compared to the *Cebpa* ^KI/KI^ controls (Figure 4A, B).

**Figure 5:**
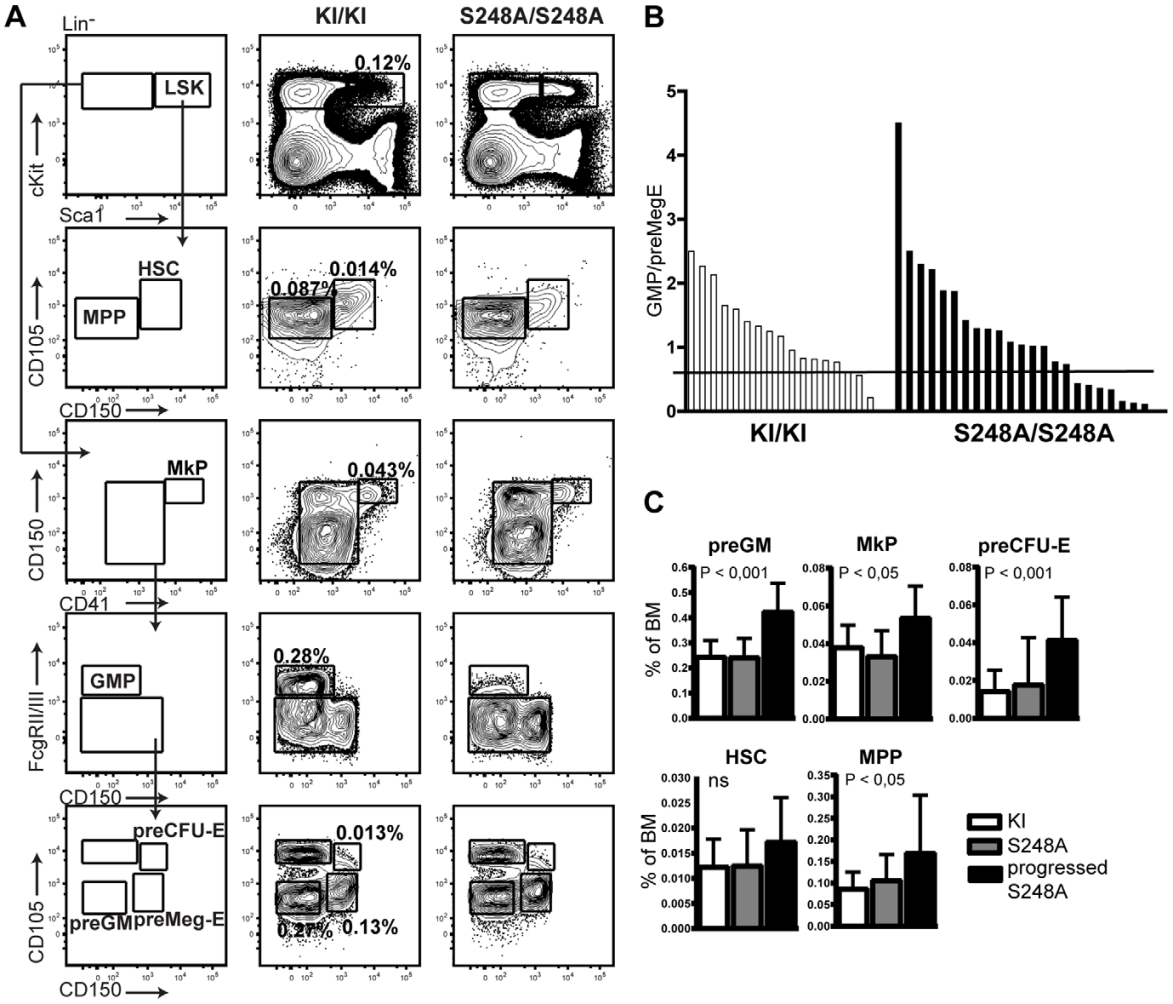
A fraction of the one-year old *Cebpa* ^S248A/S248A^ mice develops a myeloid disorder with biased lineage choice. (A) Flow cytometry analysis of the myeloerythroid progenitor compartment in BMs from *Cebpa* ^KI/KI^ (n = 17) and *Cebpa* ^S248A/S248A^ (n = 23) mice. (B) Seven out of 23 *Cebpa* ^S248A/S248A^ mice (termed “progressed”) had a skewed lineage distribution with a decreased GMP/preMegE ratio compared to *Cebpa* ^KI/KI^. Black line indicates cut-off. Cut-off was defined as mean of *Cebpa* ^KI/KI^−standard deviation. (C) Quantification of the data from (A). Numbers of mice in each of the groups were as follows: *Cebpa* ^KI/KI^ (n = 17), *Cebpa* ^S248A/S248A^ (n = 16) and progressed *Cebpa* ^S248A/S248A^ (n = 7) mice. P values designate significance between progressed *Cebpa* ^S248A/S248A^ and *Cebpa* ^KI/KI^, ns = not significant (mean +/− standard deviation).

To further delineate this apparent skewing towards the erythroid lineage and to test whether this was driven by a few *Cebpa* ^S248A/S248A^ individuals or the entire cohort, we analyzed the myeloid progenitor compartment (Figure 5A). In contrast to young mice, a subset of the one year-old *Cebpa* ^S248A/S248A^ animals displayed reduced levels of GMPs accompanied by an increase in preMegEs. Whereas 6% (1 out of 17) *Cebpa* ^KI/KI^ mice had a reduced GMP/preMegE ratio, approximately 30% (7 out of 23) of the *Cebpa* ^S248A/S248A^ animals displayed a GMP/preMegE ratio lower than the mean of the *Cebpa* ^KI/KI^ animals minus one standard deviation (Figure 5B and Figure S2). These phenotypically progressed *Cebpa* ^S248A/S248A^ mice had a significant increase in the preGMs (Figure 5C), indicating partially impaired differentiation towards the granulocyte-monocytic compartment at the preGM to GMP transition, where C/EBPα executes its lineage-instructive function [6], [7].

Furthermore, the partly impaired preGM to GMP transition was accompanied by increased levels of megakaryocytic and erythroid progenitors (preMegE, MkP, preCFU-E), suggesting that the partial block in granulocytic-monocytic differentiation directs the cells towards the erythroid lineage (Figure 5C). This lineage-skewing was also detected in colony assays, where *Cebpa* ^S248A/S248A^ BM gave rise to significantly more BFU-Es than the *Cebpa* ^KIKI^ control (Figure 4C).

In addition to the observed changes in the committed progenitor compartments, a fraction of the one year-old *Cebpa* ^S248A/S248A^ animals with biased lineage choice displayed increased frequencies of the HSC/MPP-enriched Lineage negative, Sca-1+, c-Kit+ (LSK) population compared to *Cebpa* ^KI/KI^ mice. Further resolution of this compartment showed that the progressed *Cebpa* ^S248A/S248A^ BM had a two-fold expansion of MPPs compared to *Cebpa* ^KI/KI^ controls as well as a trend towards increased numbers of HSCs (Figure 5C). Notably, we observed only a partial overlap between delayed preGM to GMP transition and expansion of the stem and multipotent progenitor compartment (Figure S2), suggesting that these events occur by different mechanisms.

**Figure 6:**
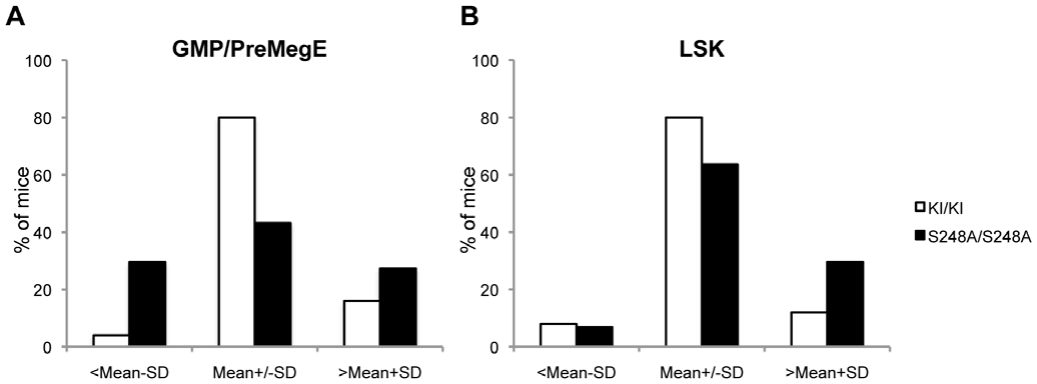
Distribution of mice based on GMP/preMegE ratio and LSK numbers. (A) Mice (age 1–2 years old) were binned based on their GMP to preMegE progenitor ratio (<mean of *Cebpa* ^KI/KI^−standard deviation; mean of *Cebpa* ^KI/KI^+/−standard deviation and >mean of *Cebpa* ^KI/KI^+standard deviation), (P = 0,012, Fishers exact test). (B) Mice (age 1–2 years old) were binned based on their level of LSK cells (<mean of *Cebpa* ^KI/KI^−standard deviation; mean of *Cebpa* ^KI/KI^+/−standard deviation and >mean of *Cebpa* ^KI/KI^+standard deviation), (P = 0,14, Fishers exact test). Total number of 1–2 year-old mice: *Cebpa* ^KI/KI^ (n = 25), *Cebpa* ^S248A/S248A^ (n = 44). See Table 1 for an overview of the phenotypes.

Next we wanted to assess in more detail whether there existed any correlations between the different aberrant phenotypes (enlarged spleen, expanded LSK compartment, erythroid-biased differentiation, etc) that we observed in old *Cebpa* ^S248A/S248A^ animals. We therefore subjected a second cohort of old animals (18–24 months) to the full experimental regime and in support of our initial findings, this group of *Cebpa* ^S248A/S248A^ animals displayed essentially the same phenotypic characteristics as the previously analyzed 1-year old cohort: Thus, whereas all (8 out of 8) *Cebpa* ^KI/KI^ mice had a phenotypical normal spleen, 29% (6 out of 21) of the *Cebpa* ^S248A/S248A^ animals displayed splenomegaly at various degrees (Figure S3C). Twenty-nine % (6 out of 21) of the *Cebpa* ^S248A/S248A^ animals had an expansion of the LSK compartment compared to 13% (1 out of 8) of *Cebpa* ^KI/KI^ (Figure S3B). Moreover, 29% (6 out of 21) of *Cebpa* ^S248A/S248A^ mice had a reduced GMP/preMegE ratio in the BM compared to 0% (0 out of 8) of the *Cebpa* ^KI/KI^ mice (Figure S3A). The phenotypes of the older *Cebpa* ^S248A/S248A^ mice are summarized in Table 1 and Figure 6. Specifically when mice are binned according to GMP/preMegE ratios, the fraction of *Cebpa* ^S248A/S248A^ animals associated with decreased GMP/preMegE ratios are significantly elevated compared to *Cebpa* ^KI/KI^ controls.

**Table 1:** Overview of the phenotypes of *Cebpa* ^S248A/S248A^ and *Cebpa* ^KI/KI^ mice.

	8 weeks		1 year		1,5–2 years	
Genotype	KI	S248A	KI	S248A	KI	S248A
Low GMP/preMegE ratio	0/6	0/6	1/17 (6%)	7/23 (30%)	0/8	6/21 (29%)
Expanded LSK compartment	0/6	0/6	2/17 (12%)	7/23 (30%)	1/8 (13%)	6/21 (29%)
Enlarged spleen	0/6	0/6	0/17	4/23 (17%)	0/8	6/21 (29%)

Importantly, the full complement of analyses performed on the 18–24 month cohort allowed us to draw a number of correlations between the different aberrant phenotypes in the old *Cebpa* ^S248A/S248A^ animals. Thus, whereas we detected a low partial overlap of mice with decreased GMP/preMegE ratio and increased LSK compartment, there was no detected correlation between mice with enlarged spleen and reduced GMP/preMegE ratio. In contrast, there was a considerable overlap of mice with expanded LSK compartment and enlarged spleen (Figure S3D–G).

At this age the phenotypically progressed *Cebpa* ^S248A/S248A^ mice had slightly lower levels of mature monocytes/granulocytes in the BM and an increased amount of mature erythroid cells in the BM compared to *Cebpa* ^KI/KI^ mice (Figure S4A). In line with this, BM cells from the progressed *Cebpa* ^S248A/S248A^ mice displayed significantly more BFU-E colonies in CFU assays, whereas the CFU-GM and CFU-GEMM were unaltered compared to BM from *Cebpa* ^KI/KI^ mice (Figure S4B).

Taken together, a fraction of old mice defective in the phosphorylation of S248 of C/EBPα exhibit a slowly developing low penetrant hematopoietic disorder(s) associated with disturbances in the myeloid compartment and/or expansion of HSCs and MPPs.

### The slowly developing hematopoietic disorders in old *Cebpa* ^S248A/S248A^ animals are cell-intrinsic

To investigate whether the above-described phenotypes are cell-intrinsic to the hematopoietic compartment, we reconstituted lethally irradiated recipients (CD45.1) with whole BM from 8 week-old *Cebpa* ^S248A/S248A^ and *Cebpa* ^KI/KI^ donors (CD45.2), and analyzed recipient BM 16 weeks post-transplantation.

**Figure 7:**
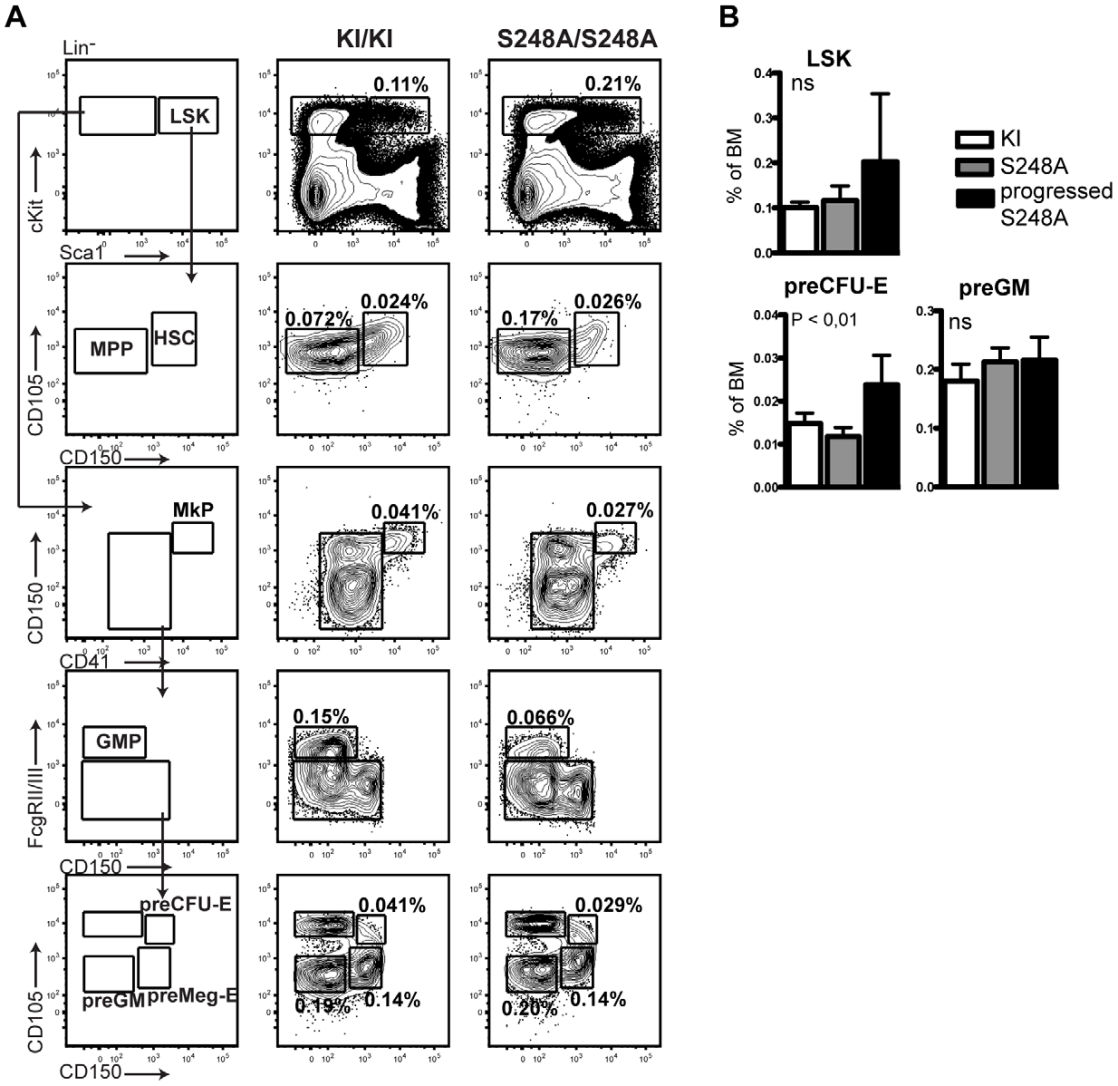
The phenotype in *Cebpa* ^S248A/S248A^ is cell-intrinsic. (A) Flow cytomtery analysis of recipients transplanted with BM from *Cebpa* ^KI/KI^ (n = 7) and *Cebpa* ^S248A/S248A^ (n = 7) mice. (B) Quantification of data from (A). Four out of seven of the *Cebpa* ^S248A/S248A^ transplanted mice displayed a reduced GMP to preMegE ratio (as defined in Figure S5) and was termed progressed. Numbers of mice in the other groups of transplanted mice were as follows: *Cebpa* ^KI/KI^ (n = 7) and *Cebpa* ^S248A/S248A^ (n = 3) mice. P values designate significance between progressed *Cebpa* ^S248A/S248A^ and *Cebpa* ^KI/KI^, ns = not significant (mean +/− standard deviation).

Similar to what was observed in one year-old *Cebpa* ^S248A/S248A^ mice, a fraction (4/7) of the recipients transplanted with *Cebpa* ^S248A/S248A^ BMs displayed diminished levels of GMPs and accumulation of megakaryocytic and erythroid progenitors (preMegE and pre-CFU-E) in comparison to recipient mice transplanted with *Cebpa* ^KI/KI^ control BM (Figure 7 and Figure S5). Consistent with the phenotype in the *Cebpa* ^S248A/S248A^ mice, there was only a partial overlap between the disturbance in the myeloid compartment and increased numbers of LSK cells (Figure S5), which supports the notion that the partly halted granulocytic-monocytic differentiation and the accumulation of HSCs and MPPs occur by two distinct mechanisms.

**Figure 8:**
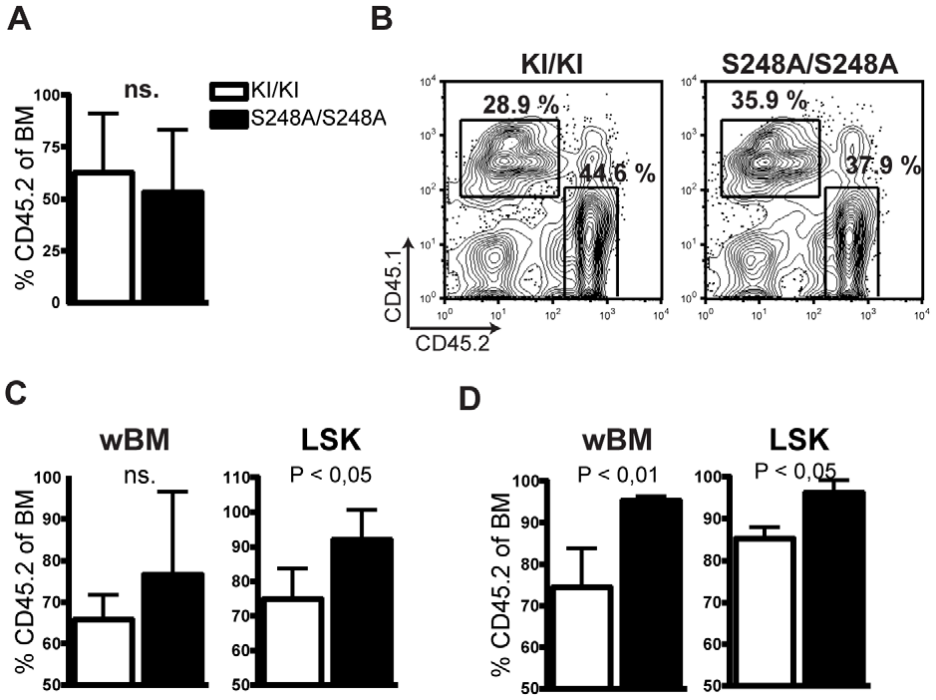
*Cebpa* ^S248A/S248A^ BM cells have a competitive advantage in comparison to *Cebpa* ^KI/KI^ BM cells. (A) Whole BM from *Cebpa* ^KI/KI^ and *Cebpa* ^S248A/S248A^ mice (CD45.2) was mixed in a 1∶1 ratio with whole competitor BM (CD45.1) and transplanted into lethally irradiated mice. BM donor contribution was assessed 18 weeks post-transplantation. *Cebpa* ^KI/KI^ (n = 7) and *Cebpa* ^S248A/S248A^ (n = 6) BM cells. (B) Input BM for the secondary transplantation show similar levels of *Cebpa* ^S248A/S248A^ and *Cebpa* ^KI/KI^ contribution in the two input samples. (C, D) BM donor contribution in whole BM (wBM) or in the LSK compartment of the secondary recipients was analyzed after 21 (C) and 34 (D) weeks. *Cebpa* ^S248A/S248A^ transplanted mice (n = 4; black bars); *Cebpa* ^KI/KI^ transplanted mice (n = 4; white bars).

To test whether the S248A allele results in a stem cell repopulation advantage or disadvantage compared to the wild type knock-in allele, we performed serial competitive BM transplantations, in which whole BM (CD45.2) from eight week-old *Cebpa* ^S248A/S248A^ and *Cebpa* ^KI/KI^ mice was mixed in a 1∶1 ratio with competitor BM (CD45.1) and transplanted into lethally irradiated CD45.1 recipients. Recipient BM was harvested 18 weeks post-transplantation and donor (CD45.2) versus competitor (CD45.1) contribution was analyzed. As shown in figure 8A, there were no significant differences in these ratios between recipients transplanted with *Cebpa* ^S248A/S248A^ and *Cebpa* ^KI/KI^ donor cells, respectively. To test whether a repopulation phenotype could be uncovered when further proliferative stress was applied to the hematopoietic system, we transplanted pooled whole BM from 6–7 primary recipients (Figure 8B) into secondary recipients, which were analyzed 21 and 34 weeks post-transplantation. At 21 weeks after secondary transplantation, the LSK compartment of mice transplanted with *Cebpa* ^S248A/S248A^ was significantly increased compared to mice transplanted with *Cebpa* ^KI/KI^ BM and this increased contribution in the stem cell compartment of *Cebpa* ^S248A/S248A^ mice resulted in a significant increase of donor BM cells at 34 weeks post-transplantation (Figure 8C, D). It should be noted that a selective advantage of donor- versus competitor cells was also observed in mice transplanted with *Cebpa* ^KI/KI^ secondary donors, since the donor/competitor ratio had also increased relative to input cells in these animals. Therefore, some selective advantage must be assigned to the genetic background of the cells used. However, the effect was significantly higher in the *Cebpa* ^S248A/S248A^ secondary recipients suggesting that the S248A mutation confer a mild selective advantage to the HSC/MPP compartment.

In summary, the *Cebpa* S248A allele results in a low-penetrant cell-intrinsic partly impairment of granulocytic-monocytic differentiation accompanied by skewing towards the megakaryocytic and erythroid compartment in a fraction of aged animals. Furthermore, these data show that *Cebpa* ^S248A/S248A^ HSCs have a competitive advantage compared to their wild type counterparts resulting in the accumulation of HSCs and MPPs in some of the *Cebpa* ^S248A/S248A^ mice.

## Discussion

Protein phosphorylation represents an important layer of cellular regulation, which may affect the stability, activity and functional property of the modified protein. Whether phosphorylation of key regulators plays a role in normal hematopoiesis has only been addressed to a limited extent and mainly in a cell culture context. The key hematopoietic regulator C/EBPα contains a number of phosphorylation sites that confer regulation under a variety of conditions [15], [29], [30]. Previously, Ras-dependent phosphorylation of C/EBPα at S248 was reported to increase its transactivational activity and promote increased granulocytic-monocytic differentiation *in vitro*, whereas Erk-dependent phosphorylation of S21 inhibited the same process [15], [17]. Furthermore, it has been shown that S21 must be dephosphorylated in order to induce not only expression of granulocytic-monocytic markers but also the erythroid-specific CD71 [16]. These findings suggest that the phosphorylation status of C/EBPα is potentially involved in regulating both granulopoiesis and erythropoiesis.

In order to extend this analysis to the proper *in vivo* context, we decided to generate mice, in which the wild type *Cebpa* gene was replaced by an allele containing a S248A substitution. Before initiating the phenotypic analysis of these animals we verified the previous reports on the requirement of S248 for *in vitro* differentiation of granulocytic-monocytic cell lines [17], [31]. Indeed, by introducing C/EBPα-ER and C/EBPα-S248A-ER into 32Dcl3 cells, we could show that the S248A variant is unable to promote myeloid differentiation. Whereas C/EBPα-ER induces the expression of myeloid markers such as Mac-1 and Gr-1 accompanied by cell cycle exit, 32Dcl3 cells expressing C/EBPα-S248A-ER fail to induce expression of these markers and continue to proliferate. This suggests that phosphorylation of S248 is required in order for the cells to differentiate into mature granulocytic-monocytic cells *in vitro*.

Given the strong requirement for S248 for *in vitro* granulocytic-monocytic differentiation, we were surprised to find that young *Cebpa* ^S248A/S248A^ mice and their relevant controls contain a similar number of mature and immature myeloid cells. In line with this, BM cells plated in semi-solid media result in outgrowth of an equal amount of colonies with similar distributions of myeloid progenitors from *Cebpa* ^S248A/S248A^ and *Cebpa* ^KI/KI^ mice. This suggests that in young mice, S248 is dispensable for the induction of granulocytic-monocytic differentiation, and consequently, that phosphorylation of S248 is neither required for the preGM to GMP transition nor for the differentiation into mature neutrophil granulocytes in mice at 8 weeks of age.

Interestingly, a fraction of older *Cebpa* ^S248A/S248A^ mice develop a myeloid disorder in which differentiation towards the megakaryocytic and erythroid lineage is promoted at the expense of granulocytic-monocytic differentiation as evident by an increase in early megakaryocytic and erythroid progenitors and a corresponding decrease in their granulocytic-monocytic equivalents. Another, partly overlapping, fraction of older *Cebpa* ^S248A/S248A^ mice presents with a mild expansion of the HSC-containing LSK compartment.

Collectively, these data contribute to the increasing body of evidence pointing towards C/EBPα as an important regulator of cell fate decisions in progenitors more primitive than GMPs. Conditional *Cebpa* knockout in the hematopoietic system in adult mice blocks the transition from CMP to GMP resulting in loss of granulocytes and monocytes but increases the numbers of myeloid blasts and MEPs [6]. Consistently, *Cebpa* ^−/−^ fetal liver contains increased numbers of erythroid progenitors and erythroid cells [32]. Furthermore, ectopic expression of C/EBPα in HSCs induces granulocyte-monocytic differentiation and inhibits erythroid development resulting in an increase in mature granulocytes and loss of megakaryocytic and erythroid progenitors [33]. In line with this, mice with mutations in the *Cebpa* allele that either reduce the transcriptional activity of the protein or destroy its growth inhibitory function, all lack mature neutrophil granulocytes and have increased numbers of erythroid cells [19], [20], [21], [23]. However, as these mice age, they progress from the neutropenic phenotype to a myeloid proliferative condition and later to an AML-like disease, showing first of all, that mutations in *Cebpa* result in a predisposition to myeloid diseases. Similarly, expression of the C/EBPα-S248A variant might act as an initiating factor in a multi-hit myeloid disorder associated with a biased lineage choice. As the development of this disorder might require additional genetic lesions this model also explains why only a fraction of the aged *Cebpa* ^S248A/S248A^ mice develop disease whereas others appear normal.

Growing evidence suggests that tight regulation of lineage-specific transcription factors plays a major role in the HSC compartment. In accordance, it has been shown that mice with various mutations in *Cebpa* have deregulated stem cell pools [19], [20], [21], [23]. The data presented in this work also support a functional role for C/EBPα in HSCs since a fraction of the 1–2 years old *Cebpa* ^S248A/S248A^ mice present with an expanded LSK compartment. Furthermore, upon serial whole BM transplantation we observed a competitive advantage of *Cebpa* ^S248A/S248A^ BM cells compared to *Cebpa* ^KI/KI^ controls and after the second round of transplantation the *Cebpa* ^S248A/S248A^ BM cells have overtaken the recipient BM. Formally, we cannot exclude the possibility that the donor-derived cells in one or more of the primary recipients had an expansion of the LSK compartment. Therefore it is unclear whether the observed increased selective advantage of *Cebpa* ^S248A/S248A^ BM cells is due to an acquired event leading to an increase of functional stem cells or an increase of self-renewal *per se* in older *Cebpa* ^S248A/S248A^ HSCs. In either case, our data demonstrates that the S248A mutation confers a competitive advantage to HSCs, when these are subjected to proliferative stress.

The development of hematopoietic disorders in older *Cebpa* ^S248A/S248A^ animals may in principle be explained by two non-exclusive models: One formal possibility is that C/EBPα-S248 is receiving input from a signalling transduction pathway that changes its activity in an age-dependent manner. Precedence for such a model comes from studies of rodent livers, where C/EBPα display different phosphorylation patterns in young and old animals. Specifically, the proportion of C/EBPα that is phosphorylated on S193 by cyclin D3-cdk4/6 increase with age, which correlates with the expression pattern of cyclin D3, and results in a reduced ability to eliminate the growth repressive potential of C/EBPα through dephosphorylation of S193 after partial hepatectomy [34], [35], [36].

Alternatively, the hematopoietic disorders in the old *Cebpa* ^S248A/S248A^ animals arise as a result of different additional genetic/epigenetic events in the *Cebpa* ^S248A/S248A^ mice, which facilitate the progression into an erythroid-biased condition, the expansion of the LSK compartment or both. We favour this second model as the *Cebpa* ^S248A/S248A^ mice with an expanded LSK compartment and an erythroid-biased lineage choice only partially overlap, which suggests that these phenotypes are driven by distinct molecular mechanisms. However, this does not exclude the possibility that mutation of S248 makes hematopoietic cells more susceptible to age-dependent changes in signal transduction pathways operating through C/EBPα.

In conclusion, our data show that S248 is dispensable for normal steady-state hematopoiesis, and that *Cebpa* ^S248A/S248A^ mice develop a low-penetrant myeloid disorder with age associated with a mild skewing towards the erythroid lineage and a partial differentiation block at the preGM to GMP transition. Additionally, *Cebpa* ^S248A/S248A^ BM display a competitive advantage during serial transplantation suggesting that phosporylation of S248 may normally serve to restrict HSC self-renewal in ageing mice.

In more general terms, the stark difference between the *in vitro* and *in vivo* phenotypes of the S248A mutant highlights the need to exert caution when extrapolating *in vitro* data to a more appropriate *in vivo* setting. Moreover, the phenotypic progression in old *Cebpa* ^S248A/S248A^ mice to a condition with a partial resemblance to the *in vitro* phenotype of mutating S248 may suggest that granulocytic-monocytic cell lines could be wired in a manner–either genetically or in terms of active signal transduction networks–that render them more relevant as models for an aged hematopoietic system.

## Materials and Methods

### Ethic statement

All mouse work was performed according to national and international guidelines and approved by the Danish Animal Ethical Committee. This study was approved by the review board at the Faculty of Health Sciences, University of Copenhagen (P10-014).

### Cell culture

All cell lines were grown at 37°C at 5% CO_2_. 32Dcl3 cells were kindly provided by A. Friedmann [24], [25]. The cells were grown in IMDM with L-Glutamine and 25 mM Hepes (Gibco) supplemented with 10% FBS (HyClone), 100 µg/ml penicillin/streptomycin (Gibco) and 1 ng/ml IL3 (Stem cell Technologies). For culturing stable transfectants 1 µg/ml puromycin (Sigma) was added. Differentiation of 32Dcl3 cells expressing ER™ fusion proteins was induced by addition of 4-hydroxytamoxifen (4-OHT, Sigma) in the presence of IL3. Phoenix-E cells (obtained from ATCC) were cultured in DMEM (Gibco) supplemented with 10% FBS (HyClone) and 100 µg/ml penicillin/streptomycin (Gibco).

### Generation of C/EBPα-expressing cells

A plasmid expressing rat C/EBPα was kindly provided by G. Behre. pBabePuro- C/EBPα-S248A-ER™ was constructed by ligating a Sfi I/EcoRI fragment containing the S248A substitution in place of the identical wild type fragment in pbabePuro-C/EBPα-ER™.

Phoenix-E cells were transiently transfected with pbabePuro-C/EBPα-ER™ or pbabePuro-C/EBPα-S248A-ER™ and virus-containing supernatant was collected after two and three days. 32Dcl3 cells were infected and selected by addition of puromycin as described in [18]. Cells were limited-diluted in 96-well culture dishes to obtain clones, which were expanded and tested for expression of C/EBPα or C/EBPα-S248A by western blotting.

### Western Blotting

Western blotting was performed as previously described [18] and probed with antibodies against C/EBPα (14AA, sc-61) and Cdk2 (Sc-163) from Santa Cruz Biotechnology.

### Cell cycle analysis

The cell cycle distribution was analyzed at day 0–3 after addition of 4-OHT as previously described [18].

### Mouse work and procedures

The Cebpa-S248A mutant was generated by using the Quickchange mutagenesis kit (Stratagene) and confirmed by sequencing. Cloning of the targeting construct, electroporation, selection of E14.1 ES cells, blastocyst injection and breeding of chimera was performed as described previously [20]. The *Cebpa* ^S248A^ and *Cebpa* ^KI^ alleles were backcrossed to C57BL/6 for at least 6 generations [20].

BM transplantations were carried out by tail vein injection of whole CD45.2 BM cells into CD45.1 recipients, which had been subjected to a lethal dose (900 rad) of gamma-irradiation 16 hours prior to transplantation. In the non-competitive setup, 1 to 2 million donor cells were used. In the competitive setup, primary transplantations were carried out by co-injecting 500.000 donor cells and 500.000 competitor cells (CD45.1). For secondary transplantations, BM cells from 6–7 primary recipients from the competitive transplantations were pooled, and 5 million of these cells were used for as secondary donor cells.

### Flow cytometry

C/EBPα-ER and C/EBPα-S248A-ER expressing cell lines were stained with Mac-1 and Gr-1 and analyzed on a FACSCalibur. Briefly, 500.000 cells were incubated with 1 µl Fc receptor block (anti-CD16/32, BD) in 20 µl PBS3%FCS on ice for 15 min. The cells were washed with cold PBS3%FCS and stained in the dark on ice with antibodies against Mac-1 and Gr-1 or corresponding isotype controls (eBioscience) for 20 min. Cells were resuspended in PBS3%FCS and run on a FACSCalibur (BD).

The hematopoietic compartment was analyzed as follows: Femurs and tibiaes were collected and crushed in PBS+3% FCS. The BM cells were stained for mature cells using antibodies against Ter119, CD71, Mac-1, Gr-1, B220, CD4, and CD8a (eBioscience) and stem and progenitor cells using antibodies against Lineage (CD3e, B220, Mac-1, Gr-1), Sca-1, c-Kit, CD105, CD41, FcgRII/III, Ter119 (eBioscience) and CD150 (Biolegend). For the analysis of transplanted animals, antibody cocktails were supplemented with CD45.1 and CD45.2 antibodies (eBioscience). After wash the mature stained cells were resuspended in PBS+3% FCS containing DAPI (0,2 µg/ml, Invitrogen) and cells stained for stem and progenitor cells were resuspended in PBS+3% FCS containing 7AAD (1 µg/ml, Invitrogen). The samples were run on a LSRII and analyzed using FlowJo software.

### Cytospins, colony assays and serial replating experiments

For the analysis of colony-forming potential, BM cells (5,000–20,000 cells/35-mm dish) were seeded in methylcellulose-based medium (M3434, StemCell Technologies Inc.) supplied with erythropoietin, IL-3, IL-6, and stem cell factor. After 10–12 d in culture, the colonies were scored as CFU-GM, BFU-E, or CFU-GEMM. In the serial replating experiments, a similar number of BM cells were seeded in M3434 medium, cultured for 7 days, and the number of colonies was counted. The cells were harvested, washed with PBS, diluted and replated in fresh M3434 medium and cultured for an additional 7 d. This procedure was repeated for 5 weeks.

Preparation of cytospins was performed as described in [21].

## Supporting Information

Figure S1 **Liver, lung and spleen tissue sections.** Tissue sections from *Cebpa* ^+/+^ and *Cebpa* ^S248A/S248A^ mice were stained with a Hematoxylin/Eosin solution. There were no changes in morphology of the tissues.
(TIF)

Figure S2 **Expanded stem and progenitor compartment in a fraction of the one-year old** ***Cebpa*** **^S248A/S248A^ mice.** (A) Seven out of 23 *Cebpa* ^S248A/S248A^ mice had a skewed lineage distribution with a decreased GMP/preMegE ratio compared to *Cebpa* ^KI/KI^. Black line indicates cut-off. Cut-off was defined as mean of *Cebpa* ^KI/KI^−standard deviation. Asterisks show the mice analyzed in figure 4B and 4C. (B) Seven out of 23 *Cebpa* ^S248A/S248A^ mice had an expanded LSK compartment compared to *Cebpa* ^KI/KI^. Black line indicates cut-off. Cut-off was defined as mean of *Cebpa* ^KI/KI^+standard deviation. Asterisks show the mice analyzed in figure 4B and 4C. (C) Partial overlap of mice with expanded LSK compartment and low GMP/preMegE ratio. (D) Enlarged spleen from a one-year old *Cebpa* ^S248A/S248A^ mouse.
(TIF)

Figure S3 **Correlation of mice with enlarged spleen, enhanced LSK compartment and myeloid-biased differentiation in 18–24 months old** ***Cebpa*** **^S248A/S248A^ mice.** (A) Six out of 21 *Cebpa* ^S248A/S248A^ mice had a skewed lineage distribution with a decreased GMP/preMegE ratio compared to *Cebpa* ^KI/KI^. Black line indicates cut-off. Cut-off was defined as mean of *Cebpa* ^KI/KI^−standard deviation. (B) Six out of 21 *Cebpa* ^S248A/S248A^ mice had an expanded LSK compartment compared to 1 out of 8 *Cebpa* ^KI/KI^. Black line indicates cut-off. Cut-off was defined as mean of *Cebpa* ^KI/KI^+standard deviation. (C) Six out of 21 *Cebpa* ^S248A/S248A^ mice had an enlarged spleen compared to *Cebpa* ^KI/KI^. (D, E, F) Quantification of the data from (A, B, C). *Cebpa* ^S248A/S248A^ mice displaying a GMP/preMegE ratio<mean of *Cebpa* ^KI/KI^−standard deviation (n = 6) were termed “progressed”. Numbers of mice in the other groups were as follows: *Cebpa* ^KI/KI^ (n = 8) and *Cebpa* ^S248A/S248A^ (n = 15) mice. Triangle designates enhanced LSK compartment, white color designates enlarged spleen, ns = not significant (mean +/− standard deviation). (G) Correlation plot of mice with enlarged spleen, expanded LSK compartment and low GMP/preMegE ratio.
(TIF)

Figure S4 **Erythroid-biased differentiation of progressed** ***Cebpa*** **^S248A/S248A^ mice.** (A) Progressed *Cebpa* ^S248A/S248A^ mice (n = 6) had increased level of erythroid cells in the BM and (B) displayed enhanced BFU-E colony formation. Numbers of mice in the other groups were as follows: *Cebpa* ^KI/KI^ (n = 15) and *Cebpa* ^S248A/S248A^ (n = 8) mice. Triangle designates enhanced LSK compartment, white colour designates enlarged spleen, P values designate significance between progressed *Cebpa* ^S248A/S248A^ and *Cebpa* ^KI/KI^, ns = not significant (mean +/− standard deviation).
(TIF)

Figure S5 **Expanded stem and progenitor compartment in some of the recipients recieving** ***Cebpa*** **^S248A/S248A^ BM.** (A) Four out of seven recipients of *Cebpa* ^S248A/S248A^ BM had a decreased GMP/preMegE ratio compared to *Cebpa* ^KI/KI^. Black line indicates cut-off. Cut-off was defined as mean of *Cebpa* ^KI/KI^−standard deviation (B) Four out of seven recipient mice with BM from *Cebpa* ^S248A/S248A^ had an expanded LSK compartment. Black line indicates cut-off. Cut-off was defined as mean of *Cebpa* ^KI/KI^+standard deviation. (C) Partial overlap of mice with expanded LSK compartment and low GMP/preMegE ratio.
(TIF)
