# Supplementary figures and images for: Phosphorylation of Serine 248 of C/EBPα Is Dispensable for Myelopoiesis but Its Disruption Leads to a Low Penetrant Myeloid Disorder with Long Latency
Source: PLoS One. 2012 Jun 8;7(6):e38841. doi: 10.1371/journal.pone.0038841 (PMC3371045; doi:10.1371/journal.pone.0038841)

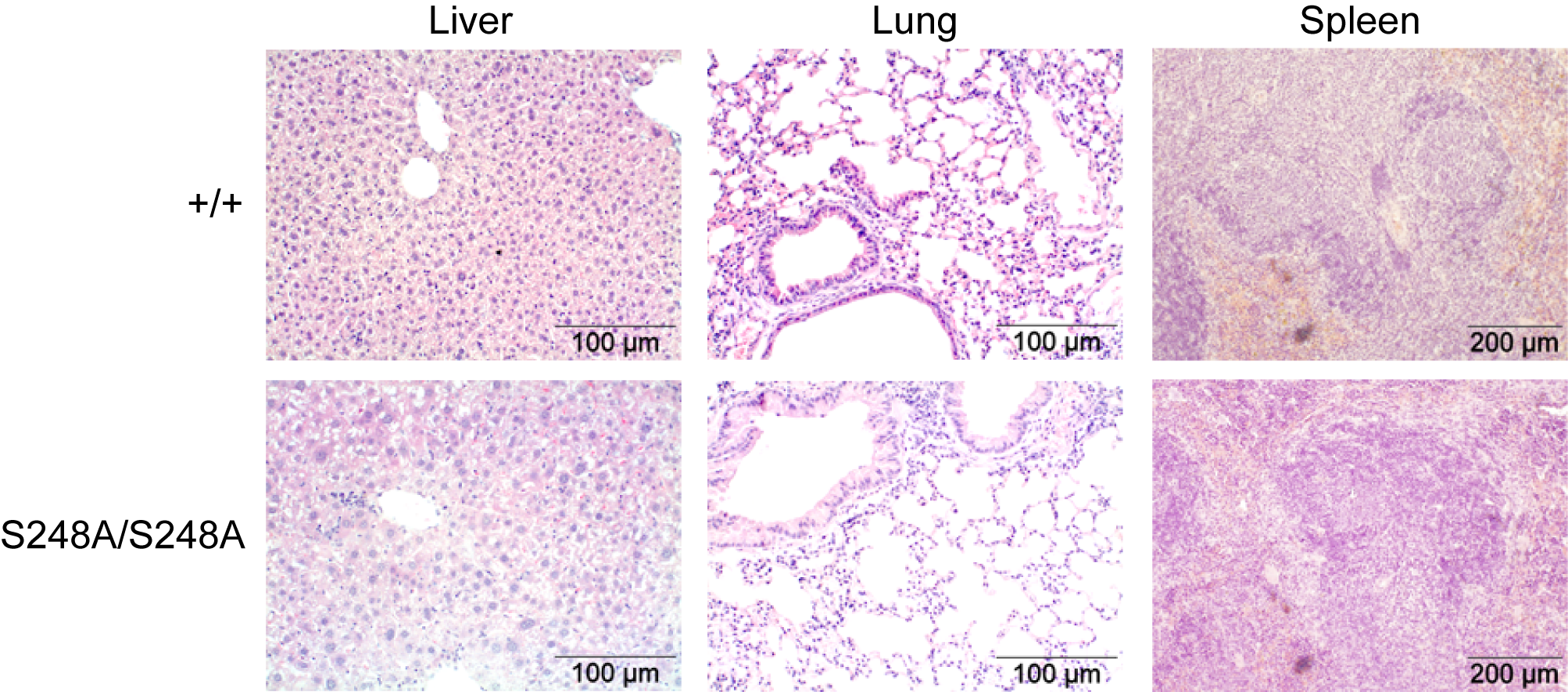

Supplement: Figure S1 — Liver, lung and spleen tissue sections. Tissue sections from Cebpa +/+ and Cebpa S248A/S248A mice were stained with a Hematoxylin/Eosin solution. There were no changes in morphology of the tissues. (TIF) [file pone.0038841.s001.tif]

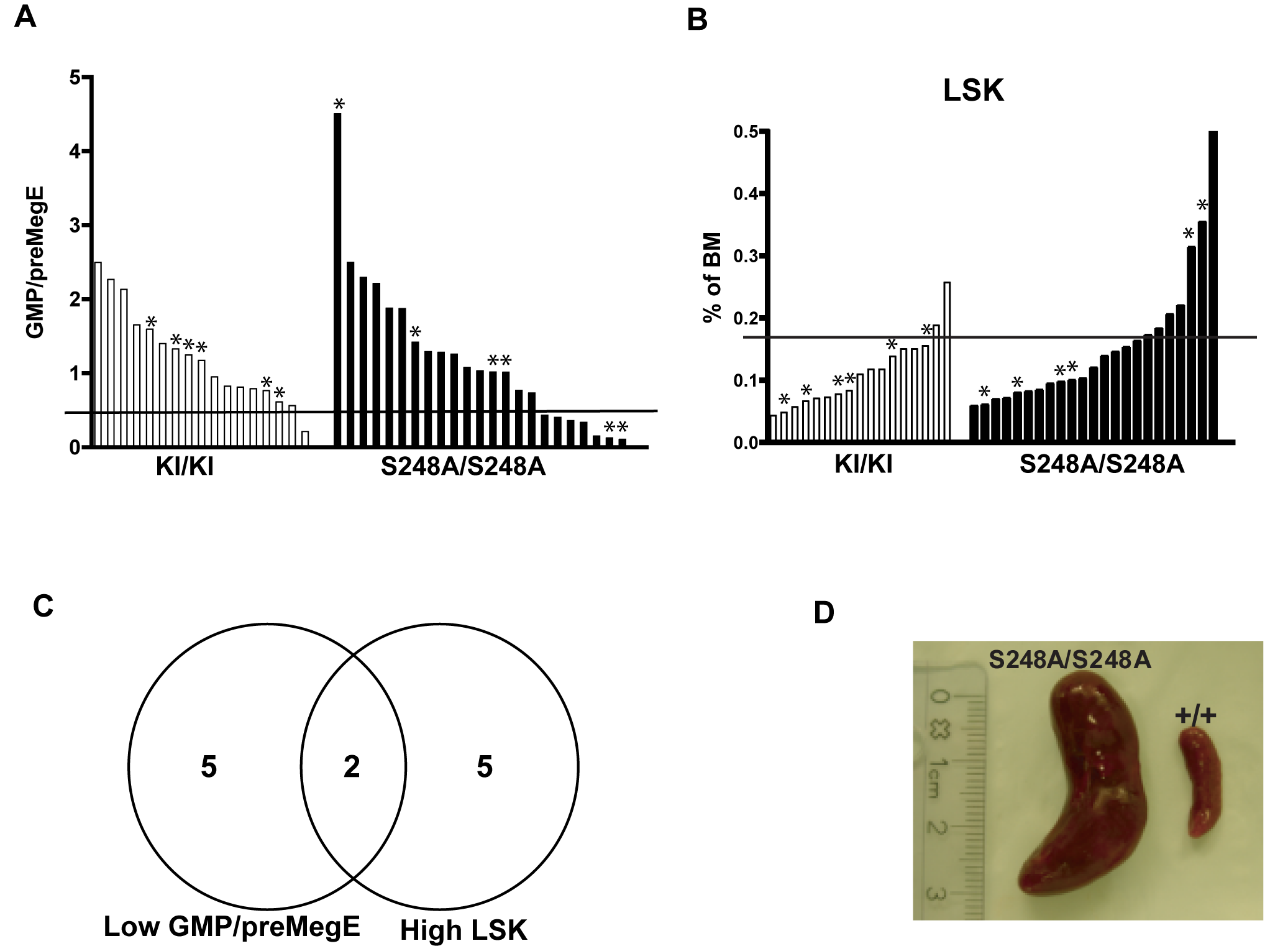

Supplement: Figure S2 — Expanded stem and progenitor compartment in a fraction of the one-year old Cebpa S248A/S248A mice. (A) Seven out of 23 Cebpa S248A/S248A mice had a skewed lineage distribution with a decreased GMP/preMegE ratio compared to Cebpa KI/KI. Black line indicates cut-off. Cut-off was defined as mean of Cebpa KI/KI−standard deviation. Asterisks show the mice analyzed in figure 4B and 4C. (B) Seven out of 23 Cebpa S248A/S248A mice had an expanded LSK compartment compared to Cebpa KI/KI. Black line indicates cut-off. Cut-off was defined as mean of Cebpa KI/KI+standard deviation. Asterisks show the mice analyzed in figure 4B and 4C. (C) Partial overlap of mice with expanded LSK compartment and low GMP/preMegE ratio. (D) Enlarged spleen from a one-year old Cebpa S248A/S248A mouse. (TIF) [file pone.0038841.s002.tif]

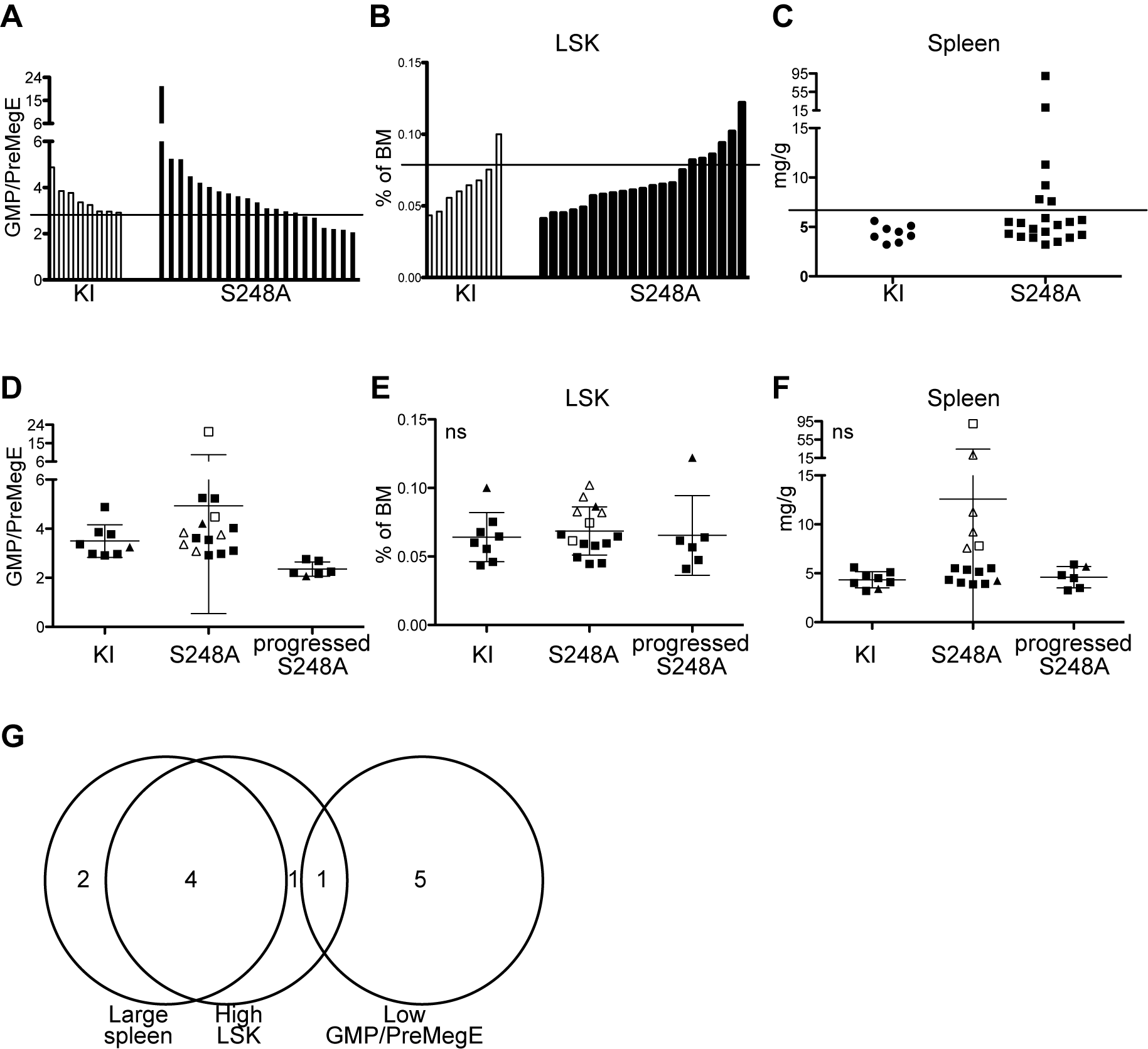

Supplement: Figure S3 — Correlation of mice with enlarged spleen, enhanced LSK compartment and myeloid-biased differentiation in 18–24 months old Cebpa S248A/S248A mice. (A) Six out of 21 Cebpa S248A/S248A mice had a skewed lineage distribution with a decreased GMP/preMegE ratio compared to Cebpa KI/KI. Black line indicates cut-off. Cut-off was defined as mean of Cebpa KI/KI−standard deviation. (B) Six out of 21 Cebpa S248A/S248A mice had an expanded LSK compartment compared to 1 out of 8 Cebpa KI/KI. Black line indicates cut-off. Cut-off was defined as mean of Cebpa KI/KI+standard deviation. (C) Six out of 21 Cebpa S248A/S248A mice had an enlarged spleen compared to Cebpa KI/KI. (D, E, F) Quantification of the data from (A, B, C). Cebpa S248A/S248A mice displaying a GMP/preMegE ratio<mean of Cebpa KI/KI−standard deviation (n = 6) were termed “progressed”. Numbers of mice in the other groups were as follows: Cebpa KI/KI (n = 8) and Cebpa S248A/S248A (n = 15) mice. Triangle designates enhanced LSK compartment, white color designates enlarged spleen, ns = not significant (mean +/− standard deviation). (G) Correlation plot of mice with enlarged spleen, expanded LSK compartment and low GMP/preMegE ratio. (TIF) [file pone.0038841.s003.tif]

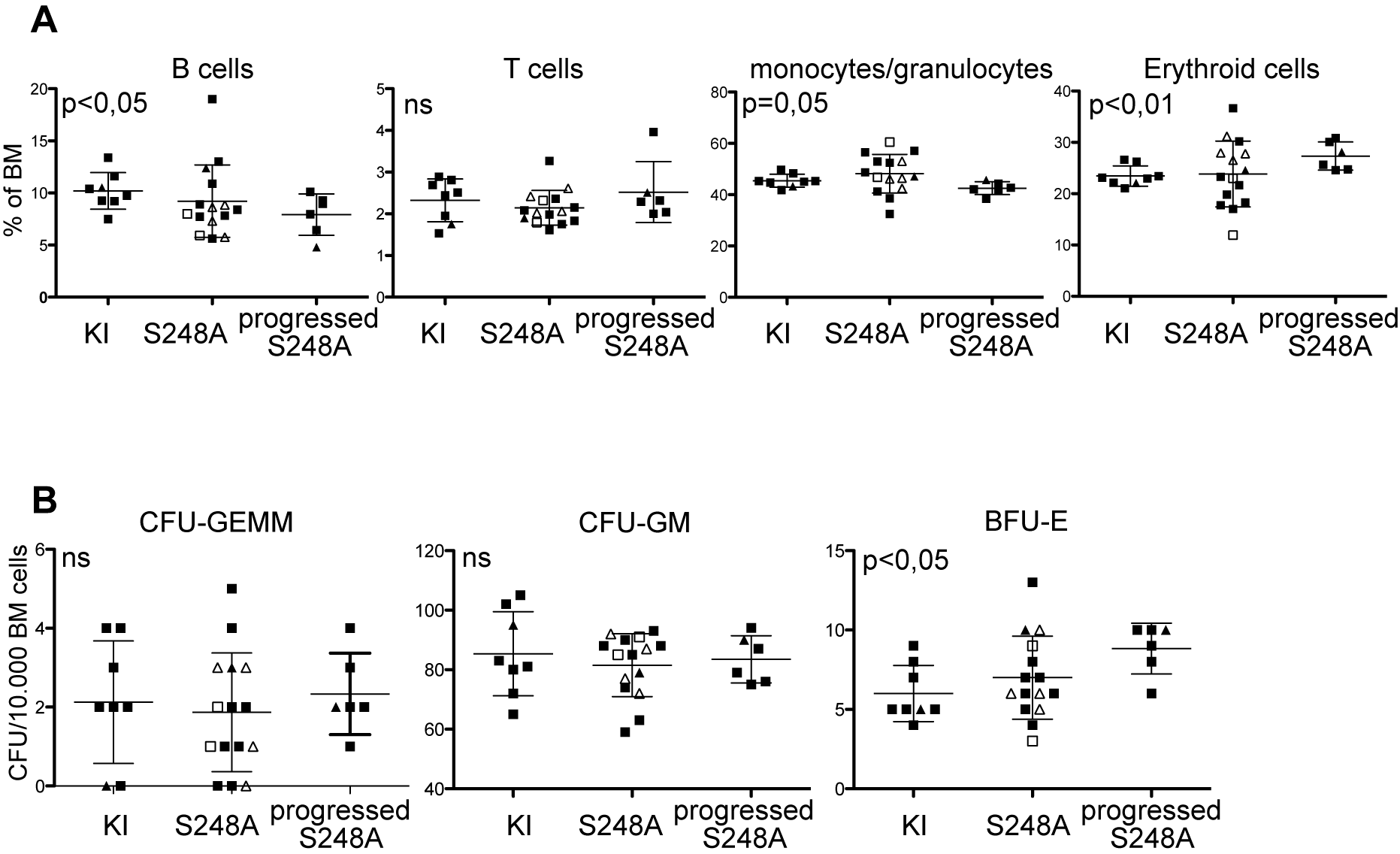

Supplement: Figure S4 — Erythroid-biased differentiation of progressed Cebpa S248A/S248A mice. (A) Progressed Cebpa S248A/S248A mice (n = 6) had increased level of erythroid cells in the BM and (B) displayed enhanced BFU-E colony formation. Numbers of mice in the other groups were as follows: Cebpa KI/KI (n = 15) and Cebpa S248A/S248A (n = 8) mice. Triangle designates enhanced LSK compartment, white colour designates enlarged spleen, P values designate significance between progressed Cebpa S248A/S248A and Cebpa KI/KI, ns = not significant (mean +/− standard deviation). (TIF) [file pone.0038841.s004.tif]

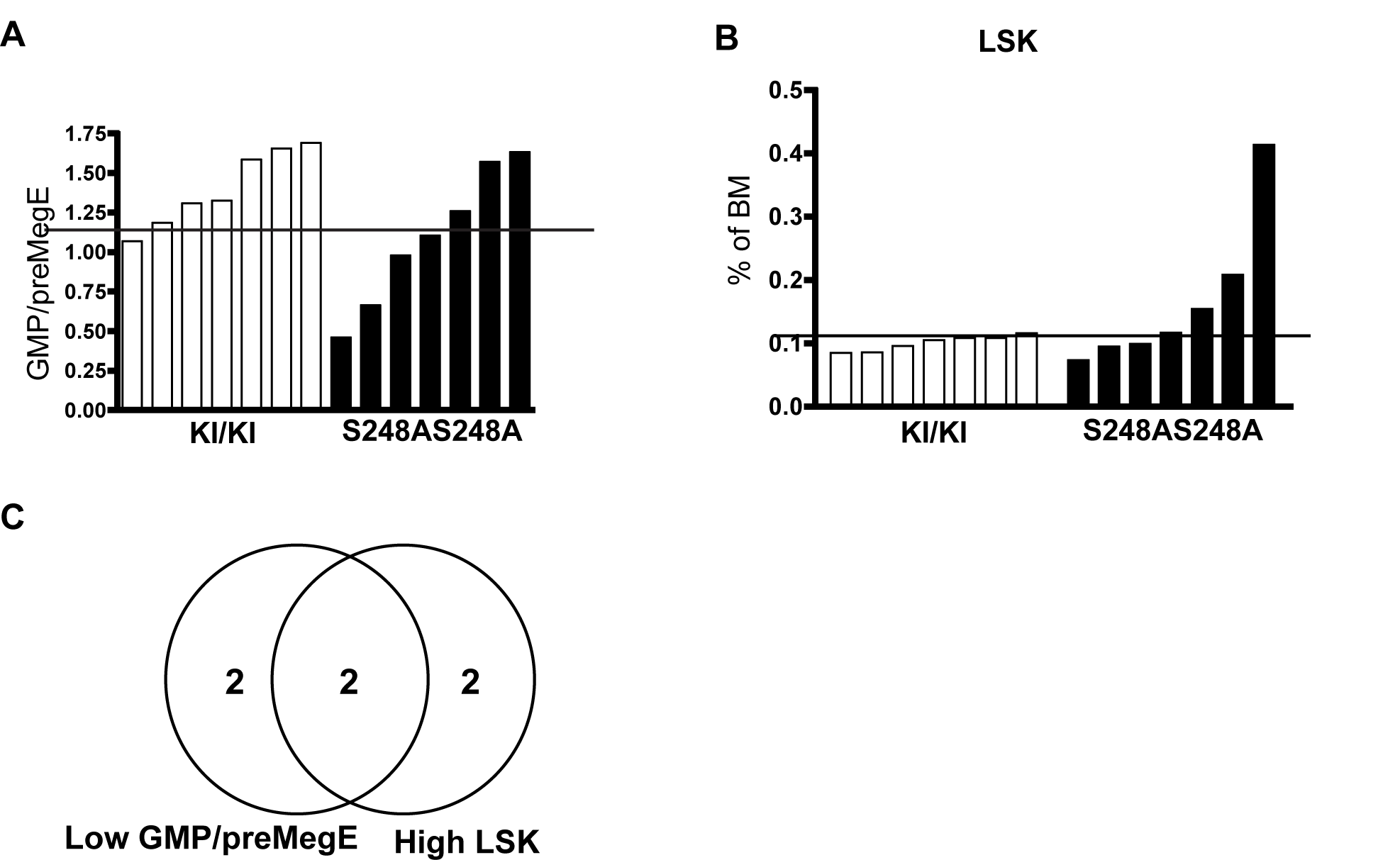

Supplement: Figure S5 — Expanded stem and progenitor compartment in some of the recipients recieving Cebpa S248A/S248A BM. (A) Four out of seven recipients of Cebpa S248A/S248A BM had a decreased GMP/preMegE ratio compared to Cebpa KI/KI. Black line indicates cut-off. Cut-off was defined as mean of Cebpa KI/KI−standard deviation (B) Four out of seven recipient mice with BM from Cebpa S248A/S248A had an expanded LSK compartment. Black line indicates cut-off. Cut-off was defined as mean of Cebpa KI/KI+standard deviation. (C) Partial overlap of mice with expanded LSK compartment and low GMP/preMegE ratio. (TIF) [file pone.0038841.s005.tif]
